# Local Corticosteroid Injection in Hand Surgery: A Review of Molecular Mechanisms, Steroid Selection, and Clinical Outcomes

**DOI:** 10.7759/cureus.117128

**Published:** 2026-09-29

**Authors:** Aneri U Patel, Leor Alkadaa, Michael J Franco

**Affiliations:** 1 Plastic and Reconstructive Surgery, Cooper Medical School of Rowan University, Camden, USA; 2 General Surgery, Cooper University Hospital, Camden, USA; 3 Plastic and Reconstructive Surgery, Cooper University Hospital, Camden, USA

**Keywords:** betamethasone, carpal tunnel syndrome, corticosteroid injection, de quervain tenosynovitis, glucocorticoid receptor, hand surgery, tenocyte, thumb carpometacarpal arthritis, triamcinolone, trigger finger

## Abstract

Local corticosteroid injection is among the most common procedures in hand surgery, yet the biologic rationale is often oversimplified as "reducing inflammation."

This narrative review summarizes the molecular biology, pharmacology, and clinical outcomes reported in the literature on corticosteroid injection for common hand and wrist conditions. The aim of this review is to bridge basic glucocorticoid biology and clinical decision-making for hand surgeons.

Glucocorticoids act through coordinated activities of both the genomic and non-genomic pathways, with varying time-related and downstream, tissue-specific effects on fibroblasts, tenocytes, and connective tissue. Steroid formulations have clinically meaningful differences with respect to particulate behavior, tissue residence, potency, solubility, and adverse effect profile. Evidence for the use of local corticosteroid injections is strongest for stenosing flexor tenosynovitis, de Quervain tenosynovitis, and mild-to-moderate carpal tunnel syndrome and more limited for limited or context-dependent for thumb carpometacarpal arthritis, Dupuytren nodules, and ganglion cysts. Adverse effects, including transient hyperglycemia, skin hypopigmentation, fat atrophy, tenocyte toxicity, chondrotoxicity concerns, and crystallization with certain anesthetic combinations, have identifiable biologic mechanisms that inform counseling and technique.

Corticosteroid injection in the hand is not a generic anti-inflammatory event but a nuanced, tissue-specific pharmacologic intervention whose outcomes depend on steroid formulation, local anatomy, disease stage, dose, injection accuracy, and patient biology. A mechanistic framework can improve patient counseling, guide steroid selection, and identify questions for future comparative trials.

## Introduction and background

As a cornerstone of hand surgery, local corticosteroid injections are frequently used for indications such as stenosing flexor tenosynovitis, de Quervain tenosynovitis, carpal tunnel syndrome, thumb carpometacarpal arthritis, wrist arthritis, Dupuytren nodules, and selected periarticular inflammatory conditions. They are often described to patients and sometimes conceptualized by practitioners as simply "reducing inflammation." However, that framing is incomplete.

Glucocorticoids are multifaceted, acting on immune cells, fibroblasts, tenocytes, synovial cells, endothelial signaling cascades, nociceptive pathways, and extracellular matrix remodeling. Their effects are tissue-specific and concentration-dependent, and clinical outcomes can vary from dramatic relief to transient improvement to limited response, depending on the diagnosis, disease stage, steroid formulation, dose, injection accuracy, and patient biology. Relevant to hand surgery are depot steroids that differ in solubility, particulate characteristics, potency, and duration of action.

The goal of this review is to bridge glucocorticoid molecular biology with practical clinical decision-making for the hand surgeon. It addresses three questions: (1) How do corticosteroids work at the molecular and cellular level in inflamed soft tissue? (2) How do available formulations differ, and does it matter which one is used? (3) What does the clinical evidence show for common hand conditions, and how should adverse effects be discussed with patients?

This narrative review was informed by literature searches of PubMed, Embase, and the Cochrane Library, with no restriction on publication date. Search terms combined "corticosteroid injection" and "glucocorticoid" with hand- and wrist-specific conditions, including trigger finger, de Quervain tenosynovitis, carpal tunnel syndrome, thumb carpometacarpal arthritis, Dupuytren disease, and ganglion cyst. Randomized controlled trials were prioritized when identifying key papers within each topic area. Once key papers were identified, the senior author (M.J.F.) performed forward and backward citation searching to identify additional relevant studies, including foundational mechanistic and pharmacologic literature not otherwise captured by the primary search terms. As this is a narrative rather than a systematic review, no formal inclusion/exclusion criteria, structured screening process, or risk-of-bias assessment was applied; systematic reviews and mechanistic studies were specifically highlighted where they addressed a clinical question not otherwise resolved by direct hand-specific trial data.

## Review

Basic science and pharmacology

Where cited evidence derives from non-hand tissue, in vitro models, or animal studies, this is noted explicitly throughout to distinguish extrapolated biologic plausibility from direct hand-specific clinical evidence.

Molecular mechanisms of glucocorticoid action

Genomic Pathway

The classical glucocorticoid signaling pathway begins with passive diffusion of the steroid across the cell membrane and binding to the cytoplasmic glucocorticoid receptor. Ligand binding induces dissociation of the receptor from its heat shock protein chaperone complex, followed by glucocorticoid receptor homodimerization and translocation into the nucleus. In the nucleus, glucocorticoid receptor dimers bind glucocorticoid response elements to transactivate anti-inflammatory genes, including annexin I and mitogen-activated protein kinase phosphatase-1. Annexin I suppresses phospholipase A2 activity and downstream prostaglandin synthesis; mitogen-activated protein kinase phosphatase-1 inactivates the p38 mitogen-activated protein kinase and c-Jun N-terminal kinase pathways that drive cytokine production [1-4].

Glucocorticoid receptor monomers also interact directly with transcription factors such as nuclear factor-kB and activator protein 1 through transrepression (Figure 1). This inhibits transcription of interleukin-1 beta, tumor necrosis factor alpha, interleukin-2, interleukin-6, interleukin-8, and interferon gamma without requiring glucocorticoid response element binding. This transrepression mechanism is central to the anti-inflammatory effect and is one reason glucocorticoid biology cannot be reduced to a single "anti-inflammatory" pathway [1-3]. Together, transcriptional activation and transrepression account for the broad anti-inflammatory effects of glucocorticoids and demonstrate that their biologic activity extends beyond activation of a single signaling pathway.

**Figure 1:**
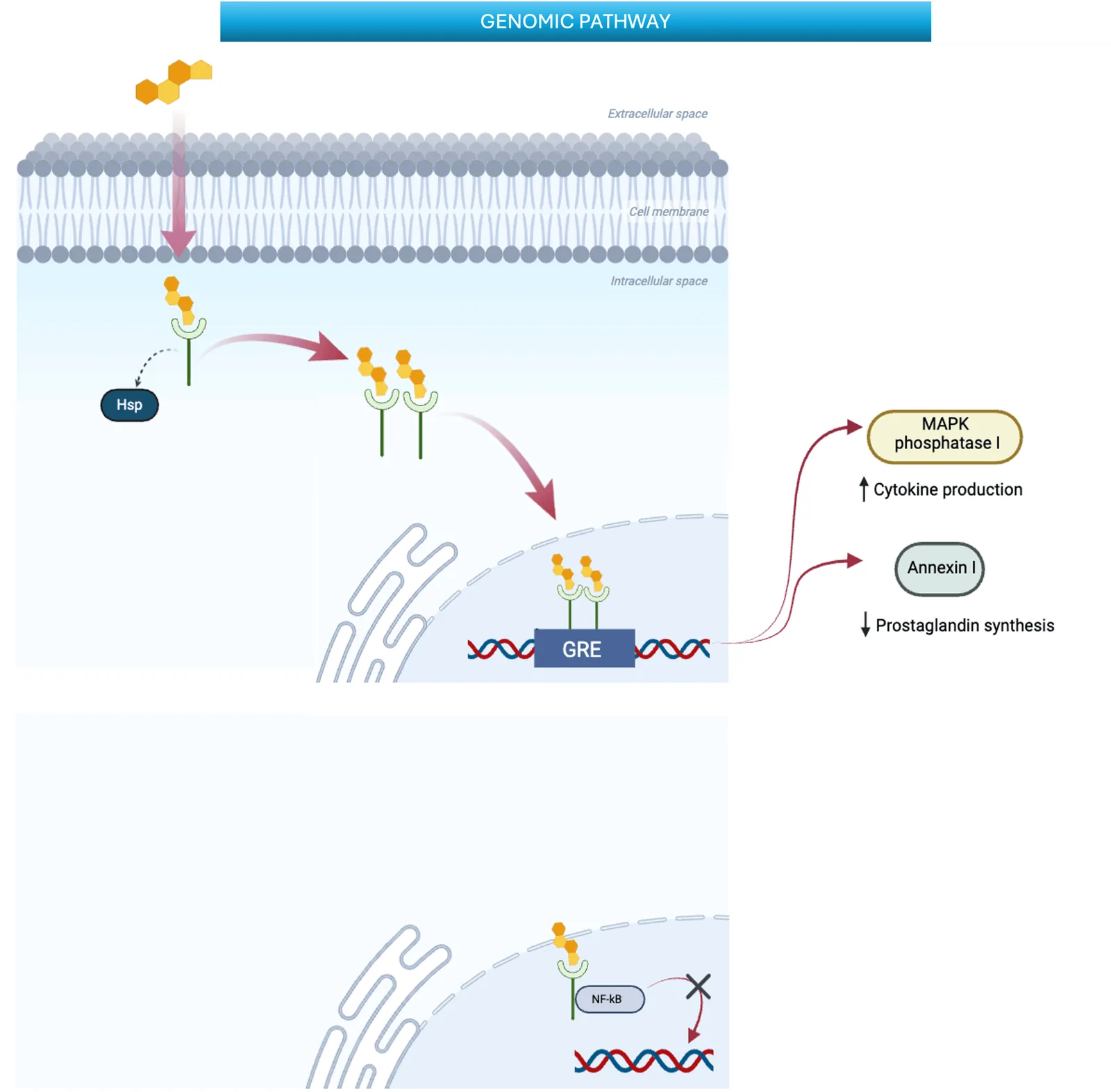
Genomic pathway. The classical glucocorticoid signaling pathway illustrating both glucocorticoid receptor dimerization and transrepression. Source: [1-4]. Created on BioRender.com

Non-genomic Pathway and Tissue Context

Patients with trigger finger, carpal tunnel syndrome, and de Quervain tenosynovitis frequently report symptomatic improvement within hours of corticosteroid injection, suggesting that mechanisms other than transcription-dependent genomic signaling contribute to early pain relief. Effects occurring too rapidly to require gene transcription - within seconds to minutes of receptor activation - are mediated through membrane-associated glucocorticoid receptor signaling, cytoplasmic kinase interactions, and direct steroid-membrane effects. These non-genomic actions may modulate vascular tone, mast cell degranulation, and acute nociceptor sensitization (Figure 2) [1,3].

**Figure 2:**
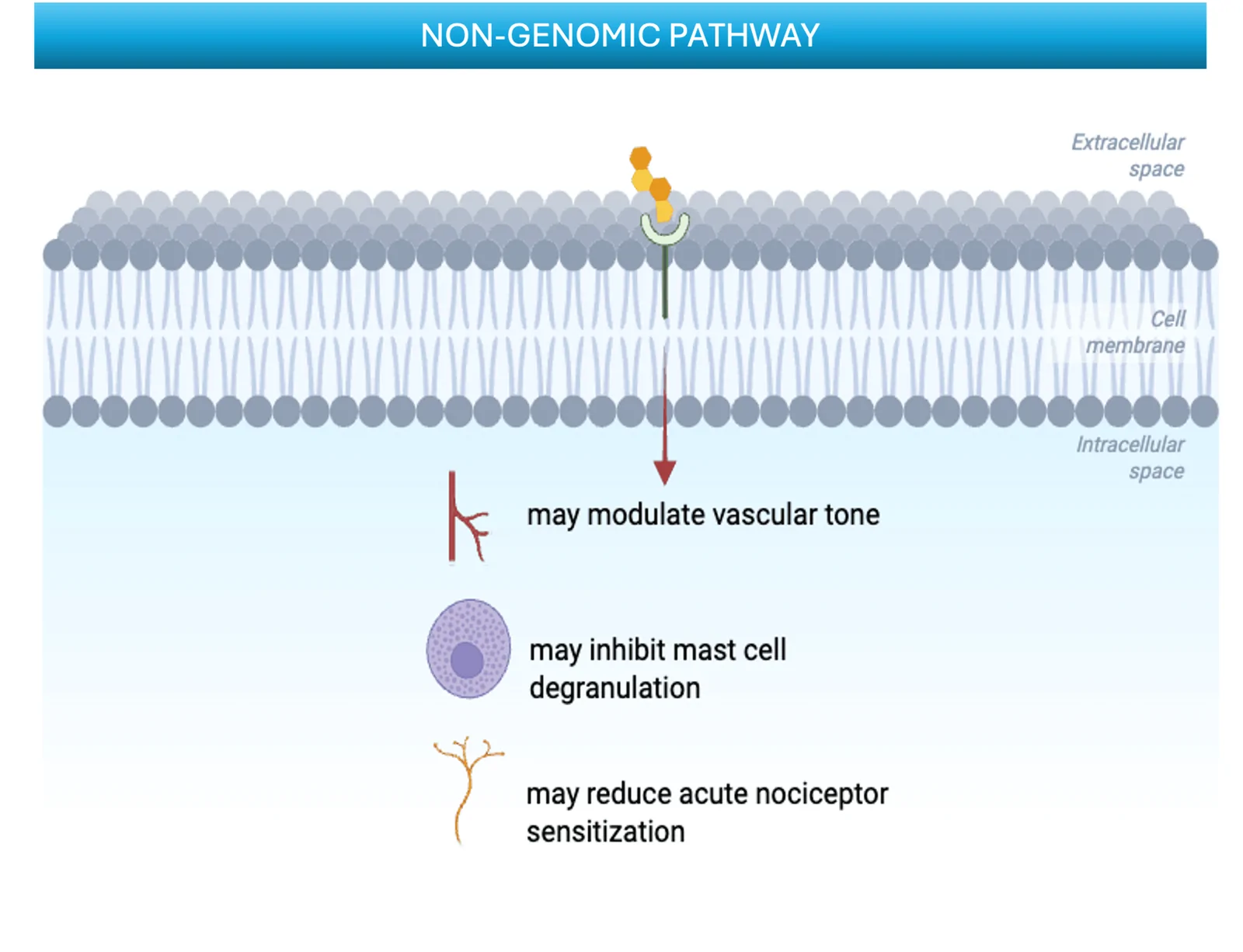
Non-genomic pathway. The non-genomic pathway of glucocorticoid signaling, occurring within seconds to minutes of receptor activation. Source: [1,3-5]. Created on BioRender.com.

Glucocorticoid effects are not uniform across cell types. Local metabolism via 11β-hydroxysteroid dehydrogenase modulates tissue-specific steroid bioavailability, and receptor isoform expression varies among synoviocytes, tenocytes, fibroblasts, and immune cells. In inflamed soft tissue, corticosteroids do not simply extinguish inflammation; they alter macrophage polarization, resolution biology, and the transition from inflammatory response to matrix remodeling. Mitogen-activated protein kinase phosphatase-1 induction has been linked to anti-inflammatory macrophage phenotypes, and glucocorticoid-induced regenerative inflammation is also shaped by macrophage metabolic signaling [4,5]. Collectively, these observations demonstrate that the clinical effects of corticosteroids are determined not only by the pharmacologic properties of the drug but also by the biologic characteristics of the tissue in which they are administered.

Effects on Tenocytes, Fibroblasts, and Connective Tissue

It should be noted that much of this evidence derives from in vitro tenocyte culture studies rather than direct clinical observation in hand surgery patients; the correlation between these cellular findings and clinically significant tendon injury in vivo remains incompletely characterized. The therapeutic benefit of steroid injection for tendon sheath disorders must be weighed against direct connective tissue effects. In vitro studies and systematic review data indicate that glucocorticoids can reduce tendon-cell proliferation and viability, decrease type I collagen synthesis, alter collagen organization, and impair tendon mechanical properties in a dose- and exposure-dependent manner [6,7]. These findings provide a biologic explanation for uncommon but important clinical concerns such as tendon rupture, delayed healing, subcutaneous atrophy, and skin changes following repeated or superficial injection. These potential complications should be discussed in the clinic during informed consent. Although no universally accepted maximum number of corticosteroid injections has been established, treatment decisions should be individualized by balancing anticipated therapeutic benefit against the potential cumulative effects on tendon and surrounding soft tissues.

The dose-response relationship is clinically important. Lower doses tend to have mainly anti-inflammatory effects, whereas higher doses may shift toward maladaptive cellular responses. For instance, high-dose methylprednisolone has been shown to activate YAP/TAZ-TEAD mechanosignaling in fibroblast-macrophage co-cultures derived from vocal fold tissue, potentially promoting fibrosis rather than resolution [8]; while this mechanism has not been directly demonstrated in hand tendon or synovial tissue, it illustrates the biologic plausibility of dose-dependent divergent effects that may extend across fibroblast populations. Conversely, prednisolone has been shown experimentally to interact with anti-fibrotic pathways in osteoarthritis-derived synovial fibroblasts [9]. This dose-dependent biology underscores the need for personalized steroid selection and dosing and advocates an individualized approach to corticosteroid use, challenging the notion that they are universally beneficial or harmful to connective tissue.

Steroid Formulations and Pharmacology

The injectable corticosteroids commonly used in hand surgery differ substantially in solubility, particulate behavior, tissue residence time, receptor-binding potency, and adverse effect profiles. These differences are clinically relevant because they influence both therapeutic duration and the risk of complications [10,11].

Triamcinolone acetonide is a low-solubility particulate suspension that achieves prolonged local tissue residence through slow crystalline dissolution. It is widely used for soft-tissue hand injection. In patients who have had injections and failed to improve going into surgery, the evidence of the triamcinolone crystals is evident. Betamethasone acetate/phosphate combines a water-soluble phosphate ester for faster onset with a less-soluble acetate component for sustained effect; particle-size studies suggest smaller particles relative to some triamcinolone preparations, which may be relevant when considering particulate behavior [10]. Methylprednisolone acetate is a particulate depot preparation with evidence in carpal tunnel syndrome and trigger finger, but it requires attention to anesthetic-mixing behavior. Dexamethasone sodium phosphate is a non-particulate, highly water-soluble solution with a shorter depot effect, making it attractive when crystallization or vascular-safety concerns are prioritized [10-13].

Mixing With Local Anesthetics

The common office practice of combining corticosteroids with lidocaine or bupivacaine deserves attention. Certain steroid-anesthetic or adjuvant-anesthetic combinations can form crystalline precipitates depending on pH, ionic concentration, and the specific agents mixed [12,13]. Much of this literature derives from research on pain and spinal injection safety, but the principle is relevant to hand surgery office practice. Injections should not be prepared far in advance; the clinician should avoid unfamiliar mixtures, and a non-particulate agent should be considered when the risk of crystallization is paramount. This represents a biologically plausible precaution extrapolated from non-hand literature rather than a hand-specific evidence-based recommendation and should be weighted accordingly in clinical decision-making.

Clinical applications: clinical outcomes by condition

Stenosing Flexor Tenosynovitis

Trigger finger represents one of the highest-yield injection indications in hand surgery. The mechanism of benefit is not purely anti-inflammatory; corticosteroids likely reduce tenosynovial volume, alter synovial signaling, and temporarily restore tendon gliding at the A1 pulley. Short-term clinical success is common, with response rates varying across published series and randomized trials based on steroid agent, dose, injection technique, symptom duration, and patient population [14-16]. Recurrence remains common enough that injection should be framed as a first-line treatment that may delay or prevent surgery rather than a guaranteed cure (Table 1).

**Table 1:** Clinical applications of corticosteroid injection in hand surgery "Strong" indicates multiple randomized trials or systematic reviews in hand-specific populations; "Moderate" indicates hand-specific comparative data of more limited scope or consistency; "Weak" indicates reliance on small case series, mechanistic studies, or evidence extrapolated from non-hand tissue. CMC: carpometacarpal; CTS: carpal tunnel syndrome; RCT: randomized controlled trial Source: [14-29]

Condition	Role of Corticosteroid Injection	Expected Benefit	Important Limitations	Key Technical Pearls	Evidence Basis
Stenosing flexor tenosynovitis	First-line treatment	May delay or prevent surgery	Lower success and higher recurrence in diabetes; not indicated for Green Grade IV (fixed contracture)	Avoid intratendinous injection	Strong (multiple RCTs, systematic review, large hand-specific retrospective cohorts)
De Quervain tenosynovitis	First-line treatment	High symptom improvement	Failure often due to incomplete compartment injection	Inject the involved first dorsal compartment; consider ultrasound for septate compartments or failed prior injection	Strong (systematic review/network meta-analysis)
Carpal tunnel syndrome	Mild-to-moderate CTS; bridge to surgery	Improves symptoms for up to ~6 months	Not appropriate for severe CTS with thenar atrophy or marked electrodiagnostic abnormalities	Ultrasound may improve safety	Strong (Cochrane systematic review)
Thumb CMC arthritis and hand osteoarthritis	Adjunct for symptom relief	Short-term pain improvement	Variable efficacy: casual and repeated injections not advised	Accurate placement important	Moderate (hand-specific RCT/meta-analysis data exist but modest effect sizes; chondrotoxicity concern extrapolated from knee literature)
Dupuytren nodules	Selected early nodules	May reduce nodule size and tenderness	No role for established cords or contracture	Intralesional injection	Weak (small clinical series and mechanistic plausibility only)
Ganglion cysts	Generally, not recommended	Little additional benefit beyond aspiration	High recurrence; skin atrophy/hypopigmentation risk	Avoid routine steroid injection after aspiration	Weak (limited comparative data)

Injection is most appropriate for Green Grade I through III disease - pretriggering, active catching, and passive/locking presentations - where the pathology remains a gliding and synovial volume problem. Grade IV disease, defined by a fixed flexion contracture of the PIP joint, reflects a structural rather than inflammatory process, and corticosteroid injection has no established mechanism to reverse it; these patients should be counseled toward surgical release rather than offered injection as a first-line option.

Diabetes mellitus is an important modifier. Diabetic patients often have lower response rates and higher recurrence [27,28,30,31], likely related to altered connective tissue biology and glycosylated matrix proteins impairing tendon collagen fiber sliding [29,32]. Comparative data between corticosteroid agents exist but yield different conclusions depending on study design and comparator. A systematic review of randomized trials found higher symptom remission with methylprednisolone compared with triamcinolone [15]. Conversely, retrospective cohort data indicate triamcinolone is associated with lower rates of subsequent reintervention than either betamethasone or dexamethasone; a single-surgeon cohort study reported significantly lower one-year failure rates with triamcinolone compared with betamethasone (16.5% vs. 34.9%; odds ratio 2.69, 95% CI 1.80-4.07) and longer time to failure (273 vs. 167 days) [26]. No single agent has demonstrated superiority across all populations and outcome measures, and agent selection may reasonably be guided by which endpoint is prioritized - short-term symptom remission versus durability of effect.

De Quervain Tenosynovitis

Corticosteroid injection is strongly supported for de Quervain tenosynovitis. The biologic rationale - reduction of synovial thickening and edema around the abductor pollicis longus and extensor pollicis brevis - is straightforward. Contemporary synthesis suggests that injection is a key component of first-line management and may be optimized by combining injection with thumb spica immobilization in selected patients [17].

The critical technical variable is accurate delivery into the involved first dorsal compartment and its relevant subcompartment. Septation of the first dorsal compartment is common, and failure to inject the occupied subcompartment accounts for an important proportion of treatment failures. Ultrasound guidance improves injection accuracy and should be considered in patients with anatomic complexity, prior failed injection, or uncertain localization [17,21].

Carpal Tunnel Syndrome

In carpal tunnel syndrome, corticosteroid injection reduces intraneural and perineural edema, decreases synovial hypertrophy within the carpal tunnel, and lowers tunnel pressure, producing symptomatic relief [19,20]. The mechanism does not reverse established axonal loss or severe demyelination. Accordingly, injection is most appropriate in mild-to-moderate disease, where it may serve as a diagnostic test, a temporizing measure while awaiting surgery, or definitive treatment in patients who are poor surgical candidates.

Patients with advanced compression neuropathy, characterized by constant symptoms, thenar atrophy, loss of discriminatory sensibility, or severe electrodiagnostic abnormalities, should not be offered injection as primary management if surgery is otherwise appropriate. The 2023 Cochrane review found that local corticosteroid injection probably improves symptoms and function for up to six months and may reduce the need for surgery at one year, but the durability of benefit is limited for many patients [20].

Thumb Carpometacarpal Arthritis and Hand Osteoarthritis

The evidence for corticosteroid injection in thumb carpometacarpal arthritis is more modest than commonly assumed. Pain relief is variable and generally short-term; benefit may depend on accurate intra-articular placement, degree of synovitis, disease stage, and concurrent splinting or activity modification [14,18]. The chondrotoxicity literature is largely derived from knee osteoarthritis trials and in vitro cartilage models, so it should not be applied uncritically to the thumb carpometacarpal joint. Still, it raises sufficient biologic concern to discourage casual or frequent repeat intra-articular injections [22,23]. Shared decision-making should include the disease stage, functional goals, the interval since the prior injection, and surgical alternatives.

Dupuytren Nodules

Intralesional corticosteroid injection into early Dupuytren nodules has been reported to reduce nodule size and tenderness in selected patients. Mechanistic work demonstrates transforming growth factor beta pathway suppression in palmar fibromatosis-derived cells, providing biologic plausibility [24,25]. This application is distinct from treatment of established cord contracture, for which corticosteroid injection has no role. Evidence remains limited to small clinical series and mechanistic studies. Prospective data or other disease-modifying strategies are lacking.

Perioperative use has also been proposed: injecting nodules preoperatively is theorized to reduce postoperative fibrosis via the same TGF-beta suppression mechanism described above, and postoperative adjuvant injection has been described in expert reviews, though dedicated comparative outcome data in Dupuytren disease specifically are lacking [33]. This mirrors the more extensively validated use of intralesional corticosteroid as an adjuvant to surgical excision in keloid and hypertrophic scar management, where combining excision with postoperative triamcinolone has been shown to reduce recurrence compared with excision alone [34].

Ganglion Cysts

Available evidence does not support corticosteroid injection as a useful adjunct to aspiration for dorsal wrist or volar ganglion cysts. Aspiration may be reasonable for selected symptomatic cysts, but recurrence is common, and corticosteroid does not appear to add durable benefit while introducing risks of skin hypopigmentation and fat atrophy at a superficial injection site [21]. For that reason, corticosteroid injection for ganglion cysts should not be presented as equivalent to the better-supported uses in trigger finger, de Quervain tenosynovitis, or mild-to-moderate carpal tunnel syndrome.

Adverse effects and patient counseling

Adverse effects of local corticosteroid injection in the hand are predictable from their biologic mechanisms (Table 2). Recognizing this biology enables more accurate patient counseling and better injection technique [22,30]. Transient hyperglycemia is the most clinically significant systemic adverse effect in many hand surgery patients. Hand-specific prospective data demonstrate that patients with higher hemoglobin A1c can experience greater blood glucose elevation after local corticosteroid injection in the hand [31]. Patients with poorly controlled diabetes or insulin-dependent disease warrant preinjection counseling, postinjection glucose monitoring, and coordination with the clinician managing their diabetes when risk is substantial.

**Table 2:** Patient counseling and technical pearls for hand corticosteroid injection CTS: carpal tunnel syndrome Source: [6,7,22,23,30-32]

Topic	Clinical Pearl
Diabetes	Counsel regarding transient hyperglycemia; lower response rates and higher recurrence.
Skin	Avoid superficial particulate steroid deposition to reduce hypopigmentation and fat atrophy.
Tendons	Never inject intratendinously; repeated injections may increase cumulative tendon risk.
Cartilage	Limit repeated intra-articular injections due to chondrotoxicity, particularly in thumb CMC and wrist joints.
Infection	Do not inject through cellulitic or ulcerated skin.
Ultrasound	Consider for de Quervain subcompartments, difficult anatomy, failed prior injection, or intra-articular injections.
Severe CTS	Refer for surgical evaluation rather than repeated injections.
Repeat injections	Reassess diagnosis and discuss diminishing benefit before additional injections.

Subcutaneous atrophy and hypopigmentation result from superficial depot deposition, especially with particulate steroids [30]. The visible impact may be greater in patients with darker skin tones, and hypopigmentation may be prolonged or permanent. Correct injection depth, avoidance of superficial steroid deposition, and selective use of smaller-particle or non-particulate agents may reduce this risk.

Tendon rupture after corticosteroid injection is uncommon but biologically plausible, given the data on tendon-cell toxicity and collagen disorganization [6,7,32]. Intratendinous injection should be avoided. Patients receiving repeated injections around tendons should be counseled that cumulative connective tissue exposure may increase risk even if the absolute rate is low.

Concerns about chondrotoxicity and accelerated cartilage loss are strongest in the knee osteoarthritis literature, but they are relevant when counseling patients about repeated injections into small joints, such as the thumb carpometacarpal or wrist joints [22,23]. Infection following injection is rare but potentially serious in the hand. Injection through inflamed, ulcerated, or cellulitic skin should be avoided. When elective surgery is anticipated, the timing of injection should be carefully considered because corticosteroid exposure near the operative field may affect infection risk and wound biology [22,30].

Special populations: pediatric and pregnant patients

Special populations also warrant separate consideration. Pediatric trigger finger is pathophysiologically distinct from the adult condition and is frequently associated with systemic or syndromic conditions; corticosteroid injection is not a standard component of its management. Nonoperative treatment with splinting or observation achieves resolution in only about 30% of cases, and surgical release - often requiring more extensive exposure than in adults - remains the definitive and considerably more effective treatment, with reported success rates exceeding 90% [35]. In pregnancy, local corticosteroid injection is generally considered a reasonable option when conservative measures such as splinting and activity modification fail, most commonly for carpal tunnel syndrome exacerbations in the third trimester; the anti-edematous, pressure-lowering effect appears to predominate over the anti-inflammatory mechanism relevant in non-pregnant patients, and injection has not been associated with adverse fetal or pregnancy outcomes when maternal systemic disease is not a contraindication, with dexamethasone often preferred [36].

Practical decision framework

A practical, clinical decision-making approach for the use of local corticosteroid injection in hand surgery begins by matching the injection indication to the strength of evidence. Among the highest-yield indications are trigger finger, de Quervain tenosynovitis, and mild-to-moderate carpal tunnel syndrome with the most favorable benefit-to-risk profiles. Those with greater uncertainty and that warrant shared decision-making are intra-articular injections for thumb carpometacarpal arthritis and wrist arthritis. Though there is biologic plausibility for Dupuytren nodule injections, outcome data are limited. Moreover, corticosteroid injection for ganglion cysts is generally not indicated as an adjunct to aspiration.

Another important aspect to consider is steroid selection. Some of this guidance rests on direct hand-specific clinical evidence, while other aspects reflect biologically plausible extrapolation from pharmacologic properties or non-hand literature, and these should be weighted accordingly. Triamcinolone remains the most widely used agent for soft-tissue hand injections and has the most direct hand-specific comparative outcome data, including the trigger finger failure-rate data discussed above. Betamethasone acetate/phosphate and dexamethasone differ in particulate behavior and solubility based on their pharmacologic properties; the preference for dexamethasone when crystallization risk is a concern is grounded primarily in spine and pain-injection literature rather than dedicated hand-specific crystallization data, and should be regarded as a biologically plausible precaution rather than a hand-proven mitigation strategy. Methylprednisolone has clinical evidence specifically in carpal tunnel syndrome and trigger finger, though comparative superiority findings vary by study design as discussed above.

Frequency, timing, and efficacy of repeat injections

The efficacy of corticosteroid injection for trigger finger declines with each subsequent injection, though repeat injections remain a reasonable option for patients preferring to avoid surgery. In a study with a minimum five-year follow-up after a single injection, long-term success ranged from 35% to 56% depending on sex and digit involvement, and 84% of eventual failures occurred within the first two years [37]. For patients who fail an initial injection, repeat injections offer diminishing but still meaningful benefit: a study of 292 repeat injections found that 39% of both second and third injections produced long-term relief, with a median time-to-failure of approximately 12 to 13 months, and roughly half of patients receiving a repeat injection achieved at least one year of symptomatic relief [38]. A large national database study similarly reported efficacy rates of 66.3%, 79.4%, and 79.6% for first, second, and third injections, respectively, suggesting continued benefit from repeat treatment even after an initial failure [39].

Regarding timing and dosing, no universally accepted minimum interval between injections has been established, though most protocols space repeat injections at least four to six weeks apart to allow adequate assessment of the initial injection's effect. A multi-facility retrospective study using low-dose triamcinolone (4 mg) without a fixed limit on the number of injections, spaced at least one month apart, reported an acceptable safety profile with no unexpected increase in adverse events [40], though repeated injections at a given site should still prompt discussion of cumulative tendon exposure, as detailed in the Adverse Effects section.

Patient factors also matter. Diabetes modifies response and risk, and darker skin tone increases the visible consequence of hypopigmentation from superficial particulate steroid deposition. Moreover, a history of multiple prior injections at the same site should prompt a more explicit discussion of cumulative connective tissue effects. Advanced compression neuropathy indicates surgical counseling rather than repeated temporizing injections.

Aside from the pharmacology, technique also drives outcomes. Injection accuracy matters for all indications but is especially important for de Quervain tenosynovitis and thumb carpometacarpal arthritis. Ultrasound guidance should be considered in complex anatomy, failed prior injection, uncertain target localization, or when precise intra-articular or subcompartment delivery is important. Intratendinous injection, prolonged pre-mixing of steroid and anesthetic, and injection through infected skin should be avoided.

Future directions

This review provides an overview of the molecular biology and considerations for the use of local corticosteroid injections in hand surgery, though several questions relevant to hand surgery practice remain inadequately studied. Comparisons of steroid formulations for trigger finger and de Quervain tenosynovitis are needed to determine whether agent selection meaningfully affects remission and recurrence. The dose-response relationship for tenocyte toxicity in vivo, and its correlation with clinical tendon injury, remains incompletely characterized. Hand-specific prospective data on glucose dynamics are limited and would be strengthened by larger cohorts with hemoglobin A1c stratification. The clinical significance of particulate steroid crystallization in hand injections, compared with the better-characterized spine and pain-injection literature, has not been directly studied. Comparative pharmacology trials examining duration of benefit, adverse effect rates, and patient-reported outcomes across steroid agents would fill an important gap in the evidence base.

## Conclusions

Local corticosteroid injections are a tissue-specific pharmacologic intervention whose effects vary and depend on steroid formulation, local anatomy, disease state, dose, injection accuracy, and patient factors. It is not a simple anti-inflammatory response. Via coordinated genomic and non-genomic mechanisms, they suppress cytokine transcription, shift macrophage phenotype, and modulate fibroblast and tenocyte behavior in ways that are therapeutically useful but potentially harmful depending on dose and context. A practical framework for their use in hand surgery - grounded in the level of evidence - supports injection for trigger finger, de Quervain tenosynovitis, and mild-to-moderate carpal tunnel syndrome. Evidence is weaker for thumb carpometacarpal arthritis and more limited conditions. Adverse effects are mechanistically predictable and often preventable through informed technique and patient selection. A mechanistic approach to office corticosteroid injection can improve counseling, reduce preventable complications, and identify which hand conditions merit future comparative pharmacology trials.
